# Genome-wide bisulphite-sequencing reveals organ-specific methylation patterns in chickpea

**DOI:** 10.1038/s41598-018-27979-w

**Published:** 2018-06-26

**Authors:** Himanshi Bhatia, Niraj Khemka, Mukesh Jain, Rohini Garg

**Affiliations:** 1 School of Computational and Integrative Sciences, Jawaharlal Nehru University, New Delhi, 110067 India; 2 National Institute of Plant Genome Research (NIPGR), Aruna Asaf Ali Marg, New Delhi, 110067 India; 3 Department of Life Sciences, School of Natural Sciences, Shiv Nadar University, Gautam Buddha Nagar, Uttar Pradesh 201314 India

## Abstract

DNA methylation is widely known to regulate gene expression in eukaryotes. Here, we unraveled DNA methylation patterns in cultivated chickpea to understand the regulation of gene expression in different organs. We analyzed the methylation pattern in leaf tissue of wild chickpea too, and compared it with cultivated chickpea. Our analysis indicated abundant CG methylation within gene-body and CHH methylation in intergenic regions of the chickpea genome in all the organs examined. Analysis of differentially methylated regions (DMRs) demonstrated a higher number of CG context DMRs in wild chickpea and CHH context DMRs in cultivated chickpea. We observed increased preponderance of hypermethylated DMRs in the promoter regions and hypomethylated DMRs in the genic regions in cultivated chickpea. Genomic location and context of the DMRs correlated well with expression of proximal genes. Our results put forth a positive correlation of promoter hypermethylation with increased transcript abundance via identification of DMR-associated genes involved in flower development in cultivated chickpea. The atypical correlation observed between promoter hypermethylation and increased transcript abundance might be dependent on 24-nt small RNAs and transcription factors binding to the promoter region. This study provides novel insights into DNA methylation patterns in chickpea and their role in regulation of gene expression.

## Introduction

Numerous cis- and trans-acting factors drive gene expression in eukaryotes^1,2^. Studies have highlighted the significance of transcription activators/repressors and non-coding RNAs in modulating various biological processes^3,4^. However, recent studies have focused on epigenetic regulation of gene expression^5,6^. Epigenetic changes refer to heritable changes in the genome without any alterations in the DNA sequence, and primarily include DNA methylation and histone modifications. DNA methylation predominantly occurs on cytosine residues. In plants, methylcytosines are found in three different sequence contexts: CG, CHG, and CHH; where H refers to A, C or T.

DNA methylation in *Arabidopsis* is mediated by seven DNA methyltransferase encoding genes: domains rearranged DNA methylase 1 (DRM1) and DRM2; chromomethylase 1 (CMT1), CMT2 and CMT3; and methyltransferase 1 (MET1) and MET2^7^. A unique feature of DNA methylation in plants is the RNA-directed DNA methylation (RdDM) pathway, which is also the predominant mode of small RNA-directed epigenetic modifications^8–10^. The presence of 5-methylcytosine (5mC) is typically associated with transposon silencing, transcriptional repression and gene silencing events. However, latest studies have demonstrated the existence of contrasting scenarios, whereby 5mCs have been linked with increased gene expression under specific biological contexts^11,12^.

Mapping of whole genome DNA methylation profile has revealed the association of methylation with various biological processes in plants, namely seed development, response to biotic and abiotic stresses, and growth and development^13–17^. Studies performed in the legume crop soybean depict the involvement of DNA methylation in various biological processes, such as response to salinity stress and small RNA abundance in cotyledons of developing seeds, to name a few^8,18^. Kim *et al*.^19^ revealed a correlation between whole genome methylation and polyploidy in soybean and common bean. Further, specific DNA methylation patterns have been speculated to be potentially responsible for phenotypic variations between different cultivars of common bean and mung bean^20^. However, despite the well-established roles of DNA methylation in several plant species, DNA methylation in chickpea has not been investigated till date.

Chickpea is a widely consumed legume crop and a rich source of human dietary protein. The analysis of various regulatory aspects of developmental processes is essential to understand their biology. Although several transcriptomic and genomic resources have been generated in the recent past^21–26^, epigenomic study in chickpea have remained elusive so far. In the present study, we examined global DNA methylation in four organs (leaves, roots, flowers and young pod) of cultivated chickpea (ICC 4958), and leaves of wild chickpea (PI 489777). Comparative analysis suggested unique methylation patterns in different organs of chickpea that can modulate gene expression. Our data depicted an atypical role of promoter methylation by highlighting association of increased transcript abundance with promoter hypermethylation. Furthermore, role of 24-nt smRNAs and transposable elements (TEs) in regulating the methylation of associated genes has been analyzed. Our results indicated possible association of smRNAs with specific differentially methylated regions (DMRs) that can drive transcription of proximal genes. The comprehensive methylome analyses performed in this study will assist in understanding the epigenetic regulation of developmental processes in chickpea.

## Results and Discussion

### Global DNA methylation in different organs of chickpea

**Figure 1:**
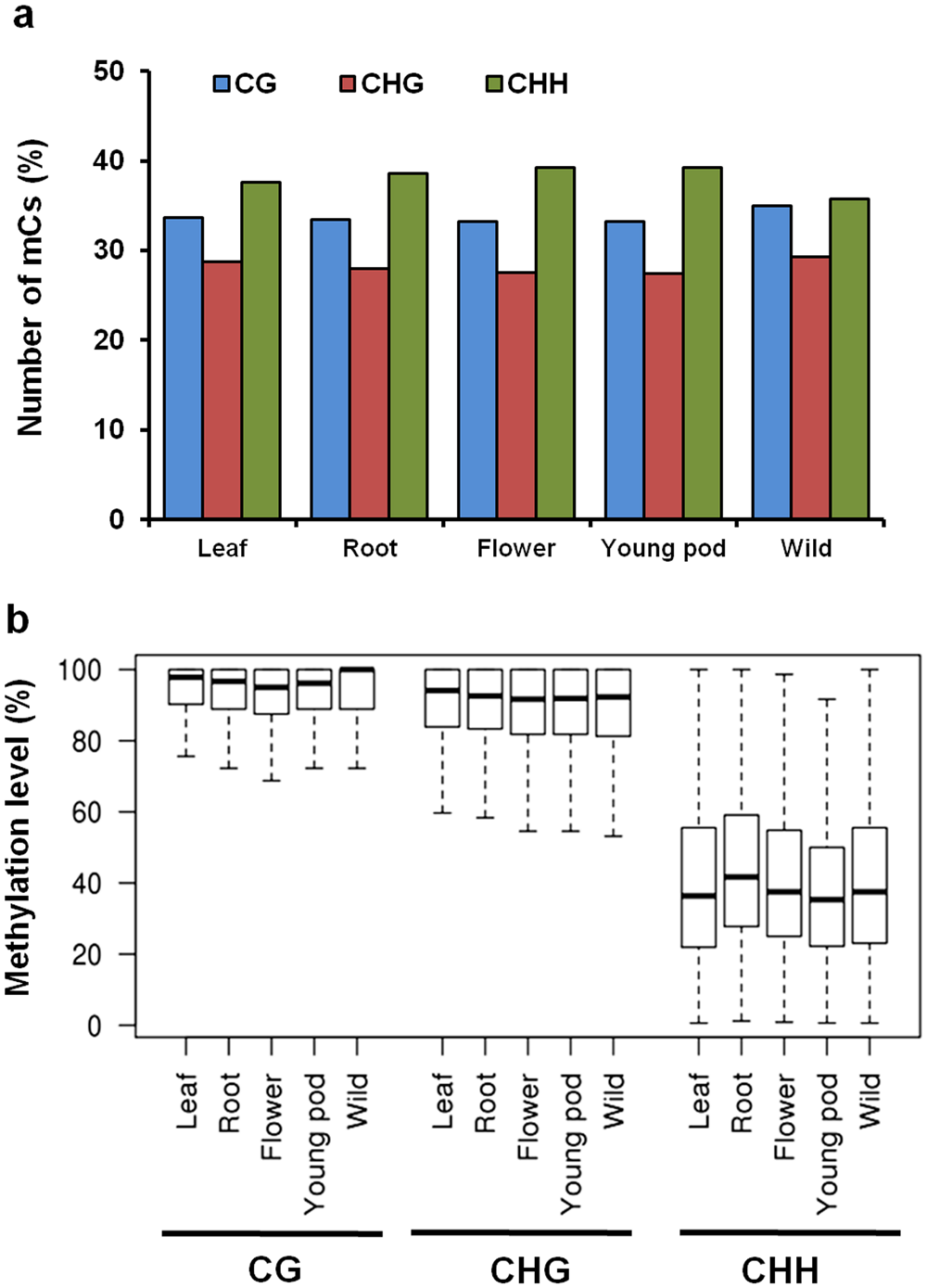
Distribution of methylcytosines in various organs of chickpea. (**a**) Percentage of methylated cytosines in each sequence context in different organs. (**b**) Box plot showing distribution of DNA methylation level in each sequence context in different organs (leaf, root, flower and young pod) of cultivated chickpea and leaf of wild chickpea.

To investigate the DNA methylation patterns in different organs of chickpea, we performed bisulfite sequencing of the genomic DNA isolated from leaves, roots, flowers, and young pod of cultivated chickpea (ICC 4958) and leaves of wild chickpea (PI 489777). Approximately 108 million high-quality read pairs were analyzed for each sample. The uniquely mapped reads provided 81–89% coverage of the chickpea genome (Supplementary Table S1), and were included in the analysis. Methylation status of each cytosine (C) residue, with a minimum coverage of 5, was determined using methylKit at *P*-value ≤ 0.0001. For all the samples analyzed, highest fraction of mCs was observed in CHH (35–39%) context followed by CG (33–35%) and CHG (27–29%) context (Fig. 1a). The average methylation level of mCs in CG context (93%) was moderately higher than CHG context (89%), but considerably higher than CHH context (38%) for all the tissues analyzed (Fig. 1b and Supplementary Fig. S1), a pattern similar to that observed in other plants^9,27,28^. Circos plots representing the chromosome-wise distribution of CG, CHG and CHH methylation in all samples are shown in Supplementary Fig. S2. Interestingly, chromosomal regions with low methylation density overlapped with high gene density regions, whereas high methylation density regions were rich in transposable elements. This indicated higher methylation of TEs as compared with protein-coding genes. This observation is in accordance with previous studies, where high levels of genomic methylation have been observed in repeats and TEs in plants for their silencing^29,30^.

### DNA methylation within gene-body and flanking regions

**Figure 2:**
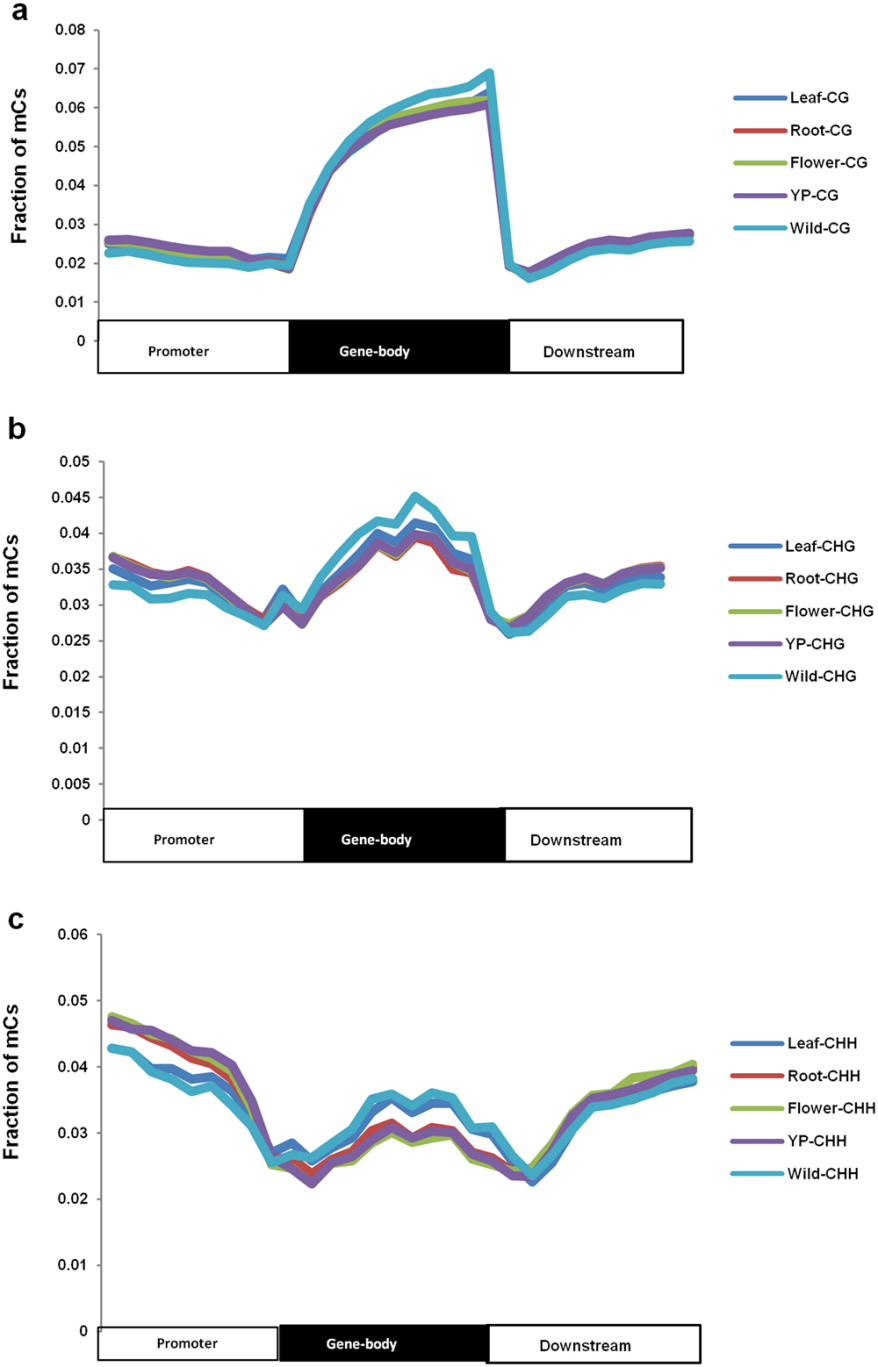
Relative fraction of methylcytosines within gene-body and 2 kb flanking (promoter and downstream) regions in each sequence context in different organs. Relative fraction of methylcytosines in CG (**a**) CHG (**b**), and CHH (**c**) contexts are shown. Each segment was divided into 10 proportionate bins to calculate relative fraction.

DNA methylation patterns were analyzed throughout gene-body and upstream/downstream regions. The fraction of mCs in CG context was highest within the gene-body, but was considerably lower in the upstream (promoter) and downstream regions for all the samples analyzed (Fig. 2a). Similar pattern was observed for mCs in CHG context with the exception that decrease in methylation density in the upstream/downstream regions was more gradual as compared with the sharp decrease observed for CG context (Fig. 2b). For CHH context, fraction of mCs was higher in the upstream and downstream regions as compared with gene-body (Fig. 2c). A noticeable decrease in the fraction of mCs was observed in the immediate vicinity of transcription start site (TSS) in CG and CHH contexts, which is expected as excessive methylation may inhibit binding of the transcriptional machinery. A tiny peak was observed at the same site for mCs in CHG context. Such abundance of methylation in proximity of TSS may be related to the nucleosome-depleted regions (NDRs) that are associated with CHG methylation^29,31^. In plants, NDRs are located in G/C-rich sequences and the NDR-associated genes are reported to be highly expressed^30^. This is primarily because nucleosome occupancy in the promoter region hinders the binding of RNA polymerase and/or transcription factors. Gene-body was enriched in CG context methylation, as has been observed in most plant species^32^. Teixeira *et al*.^32^ proposed that gene-body methylation is a by-product of transcriptional activity; activity of RNA pol II recruits the DNA methyltransferase (MET1) to the transcribed regions, which is responsible for most CG methylation across the genome. However, Takuno *et al*.^33^ proposed that body-methylated genes had lower nucleosome occupancy, evolved at a slower pace, and are more functionally important than unmethylated genes.

### Identification and distribution of DMRs

**Figure 3:**
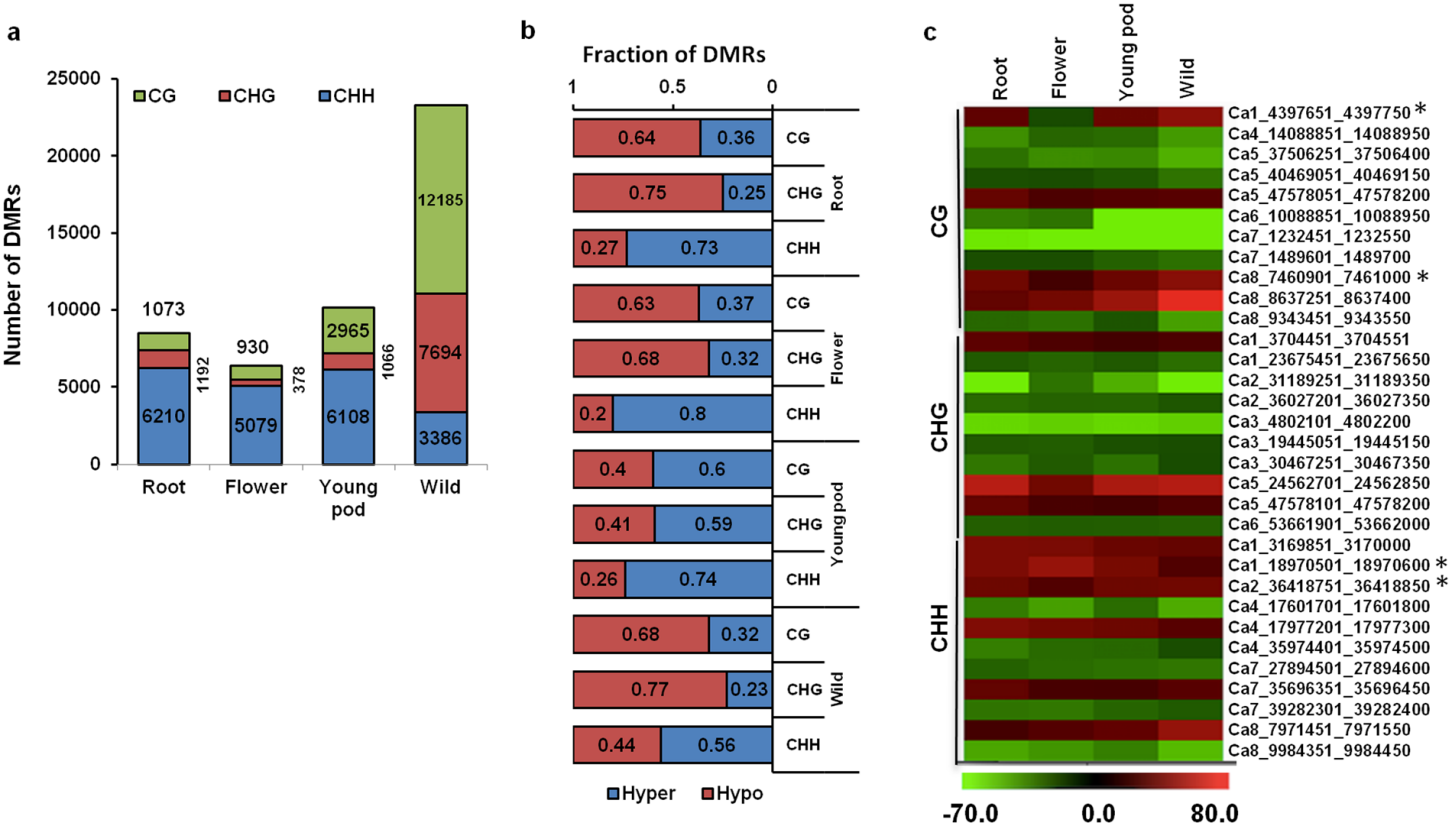
Analysis of differentially methylated regions (DMRs) across different organs. (**a**) Number of DMRs identified in different sequence contexts in various organs. DMRs in each organ were identified as compared with leaf of cultivated chickpea. (**b**) Fraction of hypo- and hypermethylated DMRs in various organs in each context. (**c**) Heatmap representing the methylation status of common DMRs among different tissues/organs. Genomic coordinates of the DMRs are given on the right side. Asterisks denote DMRs with variable methylation status in different organs. Color scale denoting the methylation status (methylation level difference) of DMRs in different organs is given at the bottom.

To identify DMRs, methylation level for each 100 bp bin of the chickpea genome was determined in each organ, and compared with that of leaf of cultivated chickpea. We identified a total of 8,475 DMRs between roots and leaves, 6,387 DMRs between flowers and leaves, and 10,139 DMRs between young pod and leaves in the cultivated chickpea (Fig. 3a). However, a significantly large number of DMRs (23,265) were identified between leaves of wild and cultivated chickpea (Fig. 3a). In all the analyzed samples of cultivated chickpea, highest number of DMRs was observed in CHH context followed by CG and CHG contexts (Fig. 3a). In contrast, highest number of DMRs was observed in CG context and lowest number in CHH context in wild chickpea (Fig. 3a).

DMRs among the samples were categorized as hypomethylated or hypermethylated based on methylation difference as compared with leaves of cultivated chickpea. A greater fraction of CG (64% and 63%) and CHG (75% and 68%) DMRs were hypomethylated, while most of the CHH DMRs were hypermethylated (73% and 80%; Fig. 3b) in roots and flowers, respectively. Interestingly, a larger fraction of DMRs in all contexts were hypermethylated (60% in CG, 59% in CHG and 74% in CHH) in young pod (Fig. 3b). Wild chickpea exhibited preponderance of hypomethylated DMRs in CG (68%) and CHG (77%) contexts, and hypermethylated DMRs in CHH (56%) context (Fig. 3b). This observation highlights overall hypomethylation in wild chickpea genome as compared with cultivated chickpea. Such variation between the cultivated and wild genotypes has been detected in previous reports as well, where genotype-specific methylation patterns were observed in different varieties of wheat, soybean and rice^34–36^. Analysis of the methylome maps of Asian cultivated rice, *Oryza sativa ssp. Japonica or indica*, and their wild relatives, *O. rufipogon* and *O. nivara*, indicated that variable methylation patterns among the varieties were a consequence of genetic diversity at DNA sequence level^34^. Gardiner *et al*.^35^ noted considerable evidence for sub-genome-specific methylation in the allohexaploid wheat with none of the genomes exhibiting selective methylation/de-methylation. Zhong *et al*.^36^ also demonstrated extensive DNA methylation in cultivated soybean (*Glycine max*) as compared with wild soybean (*Glycine soja*). Their results indicated that cultivated soybean possesses a higher level of DNA methylation than its wild counterpart, suggesting that DNA methylation increased during the domestication process.

We identified common DMR regions among all the comparisons based on their genomic coordinates. It was noted that although the methylation status of most DMRs was similar, certain DMRs exhibited different methylation pattern across the samples (Fig. 3c). For example, DMR at genomic location Ca1_4397651_4397750 was hypomethylated in flowers, but hypermethylated in other organs. Such variable methylation pattern across the organs suggested that expression of associated genes may be differently regulated in different organs via DNA methylation.

### DMRs in protein-coding genes and transposons, and their influence on gene expression

**Figure 4:**
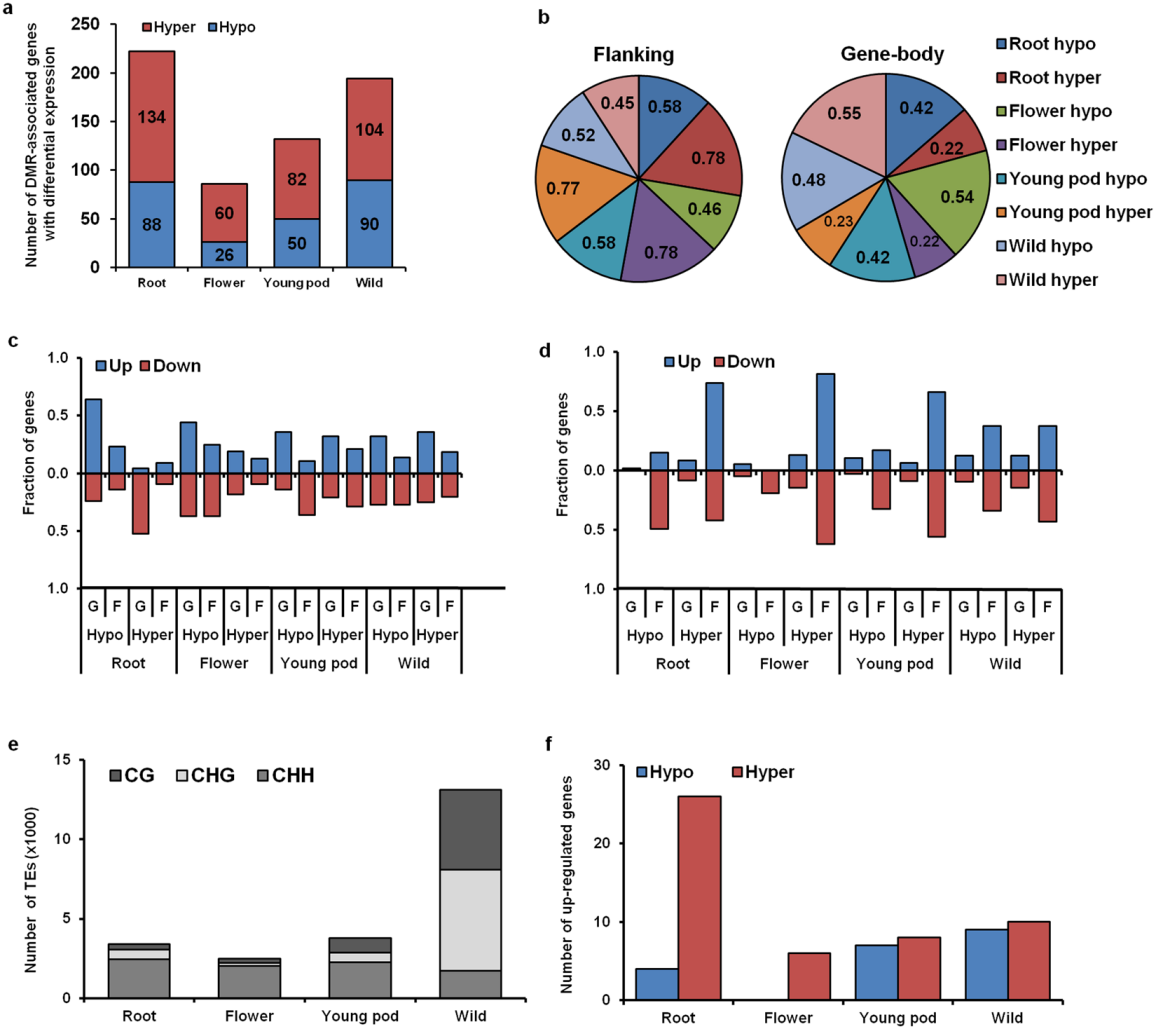
Association of DMRs with protein-coding genes and transposons, and correlation with differential gene expression. (**a**) Number of DMR-associated protein-coding genes exhibiting significant differential expression. (**b**) Fraction of gene-associated DMRs (hypo- and hypermethylated) in gene-body and flanking regions. (**c**,**d**) Fraction of up-/down-regulated genes associated with CG context DMRs (**c**) and CHH context DMRs (**d**) in gene-body (G) and flanking (F) regions in different organs. (**e**) Number of transposable elements (TEs) associated with DMRs in different contexts in various organs. (**f**) Number of up-regulated genes associated with hypo- and hypermethylated TEs in different organs.

Next, we analyzed the expression status of all the protein-coding genes in various organs. A total of 8,150 genes in roots, 7,919 genes in flowers, 8,228 genes in young pods and 7,007 genes in wild chickpea leaves were found to be up-regulated; while 6,879 genes in root, 6,598 genes in flowers, 5,989 genes in young pods and 7,204 genes in wild chickpea leaves were observed to be down-regulated (Supplementary Fig. S3). Of all protein-coding genes in chickpea, 4,011 genes in root, 2,933 genes in flower and 5,300 genes in young pod were found to be associated with DMRs (Supplementary Fig. S3). In wild chickpea, 11,438 protein-coding genes were associated with DMRs (Supplementary Fig. S3). The DMR-associated genes were further analyzed to correlate differential methylation with differential gene expression. Approximately 222, 86 and 132 DMR-associated genes in roots, flower and young pod, respectively, and 194 DMR-associated genes in wild chickpea, exhibited significant differential expression, suggesting that DMRs might regulate their expression (Fig. 4a). Among these, a greater number of DMR-associated genes (142 in roots, 54 in flower and 80 in young pod) were up-regulated in cultivated chickpea, while a higher number of genes (108 genes) were down-regulated in wild chickpea (Supplementary Fig. S3). Further, a significantly higher number of genes were associated with hypermethylated-DMRs than hypomethylated-DMRs in all the samples analyzed (Fig. 4a). These hypermethylated-DMRs were predominantly located in the upstream and downstream regions in cultivated chickpea, whereas such distribution of DMRs was not observed in wild chickpea (Fig. 4b). The differential expression of only a small fraction of the DMR-associated genes has been reported in previous studies too^9,37,38^ which suggests that DNA methylation can influence gene expression directly or indirectly via affecting the binding of transcriptional regulators.

Next, we examined the effect of methylation context (CG or CHH), and genomic location (gene-body or flanking) of the DMRs on transcript abundance (Fig. 4c,d). In all the organs of cultivated chickpea, a significant fraction of up-regulated genes harbored hypomethylated CG context DMRs within gene-body (64% in root, 44% in flower, and 36% in young pod; Fig. 4c), or hypermethylated CHH context DMRs in the promoter/downstream region (74% in root, 81.6% in flower, and 66% in young pod; Fig. 4d). This suggested a positive regulation of gene expression by promoter/downstream CHH context DMRs in cultivated chickpea. On the other hand, CG context DMRs within gene-body regulated gene expression negatively. Such a distinct pattern was not observed for differentially expressed genes in wild chickpea (Fig. 4c,d). Our observations were in accordance with previous studies^11,34,39^. For example, Wan *et al*.^39^ observed that DMRs correlated positively with gene expression were enriched in the promoter region. Multiple transcription factor-binding motifs were enriched within the promoter DMRs that associated positively with gene regulation. It was suggested that binding of transcription factors to these promoter DMRs might induce transcription of associated gene(s)^39^. Halpern *et al*.^40^ demonstrated that *FoxA2* gene promoter is methylated preferentially in tissues showing its high expression. Using gene reporter assays, they showed that the unmethylated promoter region correlated negatively with gene expression, supporting the notion that methylated sequences block binding of repressor protein molecules^40^. The correlation between DNA methylation and transcription of target genes has been analysed in detail by Zhu *et al*.^11^. While the traditional view of protein-DNA interactions involves transcription factors (TF) binding to unmethylated DNA motifs, Zhu *et al*.^11^ discuss binding of proteins to methylated DNA sequences. It was proposed that DNA methylation creates new binding sites for TFs or that TFs may recognize different sequences with or without DNA methylation^11^.

Our results suggested that gene expression was dependent on methylation context (CG and CHH) and the genomic location of DMRs (gene-body and promoter/downstream) in cultivated chickpea. This is consistent with previous studies that also highlighted the significance of genomic location and context of methylation in modulating gene expression under certain conditions^37,38^. It has been hypothesized that the binding of specific transcription factors (TFs) to DNA may be dependent on its methylation status, with certain TFs recognizing different sequences with or without DNA methylation^11,41–43^. Another possibility is the binding of specific proteins (methylated-sequence binding proteins), which further compete with TFs for the same DNA sequence. In addition, gene expression may be controlled by the organ-specific expression of TFs/DMR-binding proteins, adding another layer to the complex regulation of gene expression. Such scenarios probably modulate gene expression in a DMR-dependent manner and might be responsible for the differential gene expression in different organs.

We investigated the role of DMRs in methylation-mediated transposon silencing. We determined the number of TEs associated with DMRs in each context for each sample. The highest number of TEs was found to be associated with CHH and CHG context DMRs in cultivated and wild chickpea, respectively (Fig. 4e). Analysis of the associated genes revealed that a significant number of up-regulated genes were associated with hypermethylated TEs in roots (26 out of 30 genes) and flowers (all 6 genes) (Fig. 4f). Such a pattern was not seen in young pod or wild chickpea. In roots, 10 of the 26 genes had TEs in downstream flanking region with CHH DMRs within TE body, highlighting the role of CHH DMRs in flanking regions. Thus, we speculate that TE hypermethylation may be a crucial factor in up-regulation of associated genes in roots and flowers through the involvement of methylation-associated regulatory proteins^11^.

### Functional categorization of DMR-associated genes

**Figure 5:**
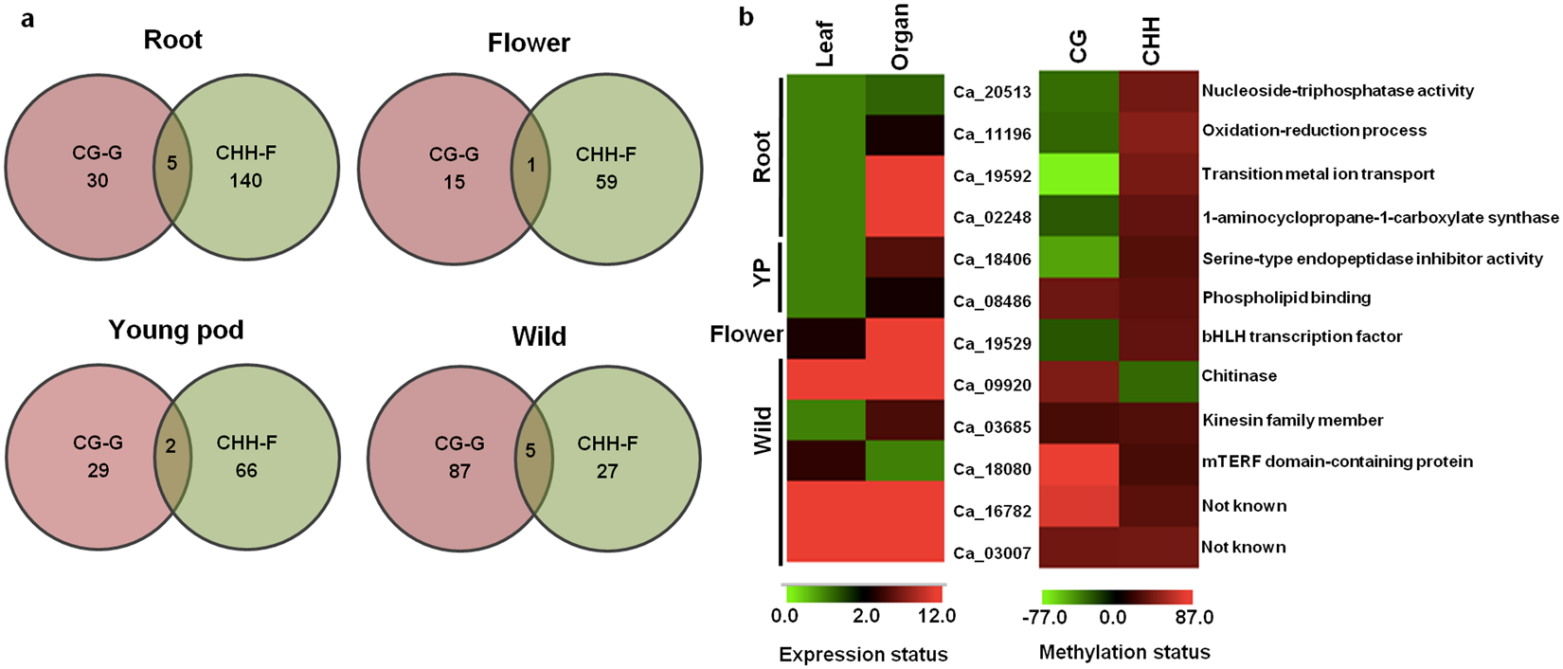
Analysis of expression and methylation status of genes with gene-body (CG) and flanking (CHH) DMRs in various organs. (**a**) Venn diagram representing genes common between CG-G and CHH-F DMRs in root, flower and young pod of cultivated chickpea, and leaf of wild chickpea. (**b**) Heatmaps depicting the expression (left) and methylation status (right) of common genes from Venn diagram. Left heatmap: color scale at bottom denotes fold change of genes in different organs; right heatmap: color scale denotes methylation status of CG and CHH DMRs in different organs.

Our above observations point towards a critical role of location and context of the methylated region in modulating gene expression. It is expected that such regulation might influence the expression of genes involved in organ development. Hence, we analyzed the DMR-associated genes in greater detail. A total of 30, 15, 29 and 87 genes in roots, flowers, young pod and wild chickpea, respectively, were found to be associated with CG context DMR in gene-body only (Fig. 5a, Supplementary Table S3). In addition, a total of 140, 59, 66 and 27 genes in roots, flowers, young pod and wild chickpea, respectively, were associated with CHH context DMR in flanking regions only (Fig. 5a, Supplementary Table S3). To analyze the role of multiple DMRs in regulating organ-specific gene expression, genes common to both categories (CG-gene-body and CHH-flanking) were identified for each organ (Fig. 5a). We obtained five such genes common in case of roots, one in flower, two in young pod and five in wild chickpea (Fig. 5a). One gene with differential expression and methylation in flower, Ca_19529, showed homology with two basic helix-loop-helix transcription factors, BIGPETAL (BPE) and CIB2, which have been reported to be involved in the regulation of petal size and flower development in *A. thaliana*^44,45^. Ca_19529 was found to be significantly up-regulated (fold change = 14; Fig. 5b) in flower and was associated with two DMRs, a CG hypomethylated-DMR in gene-body and a CHH hypermethylated-DMR in promoter region (Supplementary Table S2). However, this gene was not associated with any DMR in other organs (Supplementary Table S2). Thus, we speculate that the DMRs associated with Ca_19529 may be responsible for the flower-specific up-regulation of this gene.

**Figure 6:**
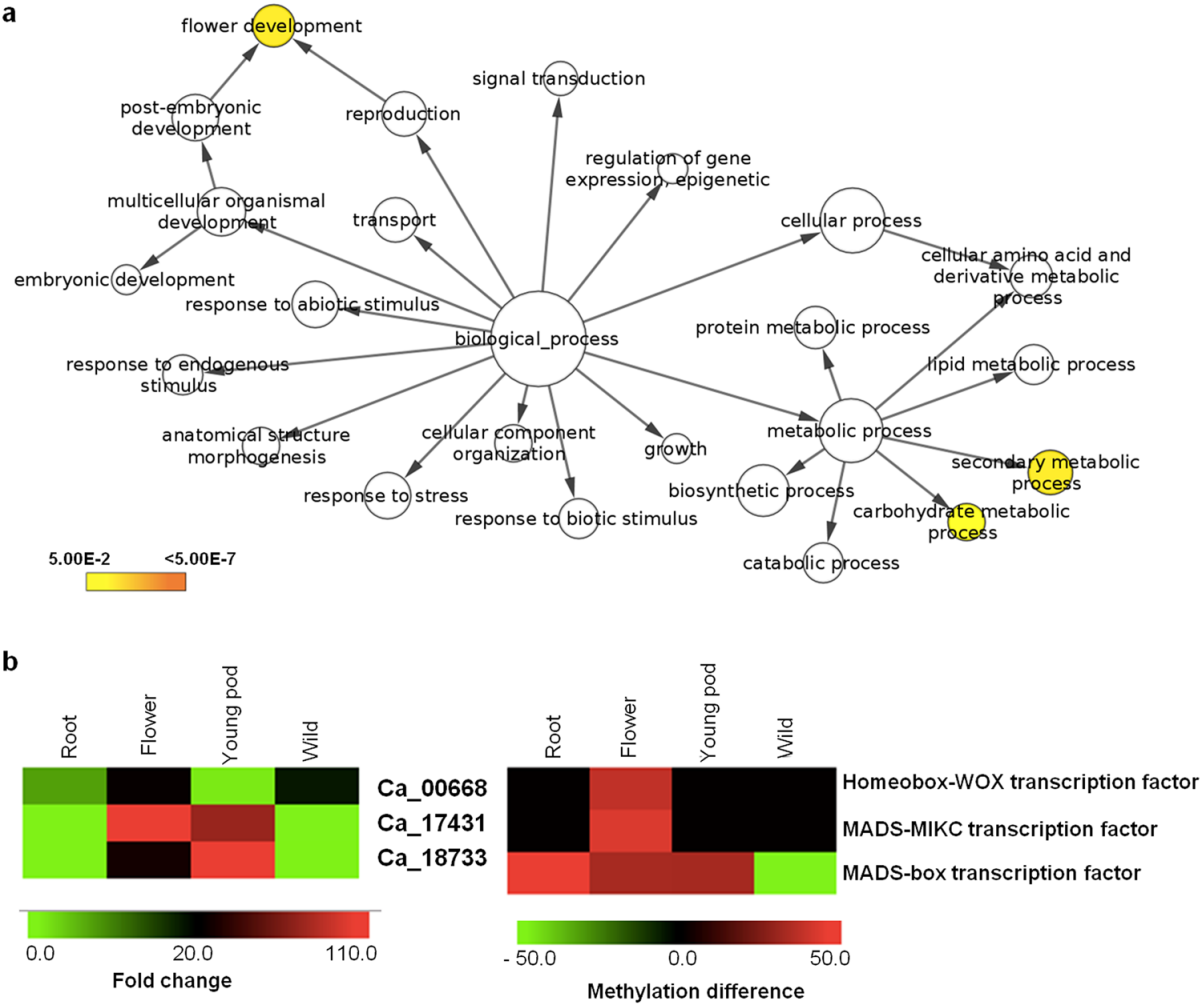
GO enrichment of hyper-methylated genes in flowers and different expression/methylation of genes involved in flower development. (**a**) GO Slim enrichment analysis of hypermethylated-DMR-associated genes in flower. The significantly enriched GO terms are highlighted in yellow color (p-value < 0.05). (**b**) Heatmaps depicting the expression pattern (left) and methylation status (right) of genes involved in flower development. Left heatmap: color scale represents fold change of genes in different organs; right heatmap: color scale denotes methylation status genes in different organs.

Gene enrichment analysis of hypermethylated genes in flower revealed “flower development” to be a significantly over-represented GO term (Fig. 6a). The genes belonging to flower development process (*Ca_00668*, *Ca_17431* and *Ca_18733*) were up-regulated and were found to be associated with hypermethylated CHH DMRs in their flanking region in flower only (Fig. 6b; Supplementary Table S2). Here also, we noticed that gene expression was positively correlated with flanking region methylation in CHH context, which supported our above observations. Such correlation between up-regulated genes and promoter methylation was not observed for other organs, suggesting that promoter methylation was a critical factor driving the flower development pathway. The DMRs in promoter regions of both genes possess binding sites for multiple transcription factors, including TCP, MADS, TALE, C2H2, GATA, and MYB in case of Ca_18733, and ERF in Ca_19529. Some of these transcription factors have been reported to bind DNA in a methylation-specific manner^11,41^. Thus, our results reveal an important role of promoter hypermethylation in the chickpea genome. Previous studies also demonstrated that other epigenetic factors, such as histone modifications, regulate multiple downstream genes/proteins involved in flower development. For example, H3K27me3, a repressive histone mark catalysed by PRC1 and PRC2 in *Arabidopsis*, was found to be essential for floral organogenesis^46^. In rice, H3K27me3 has been reported to regulate flowering time^46^. Another histone modification marking active chromatin, H3K36me3, also regulated floral organ development^46^. Therefore, it seems that various epigenetic factors work together to regulate flower development, which needs to be investigated in detail.

**Figure 7:**
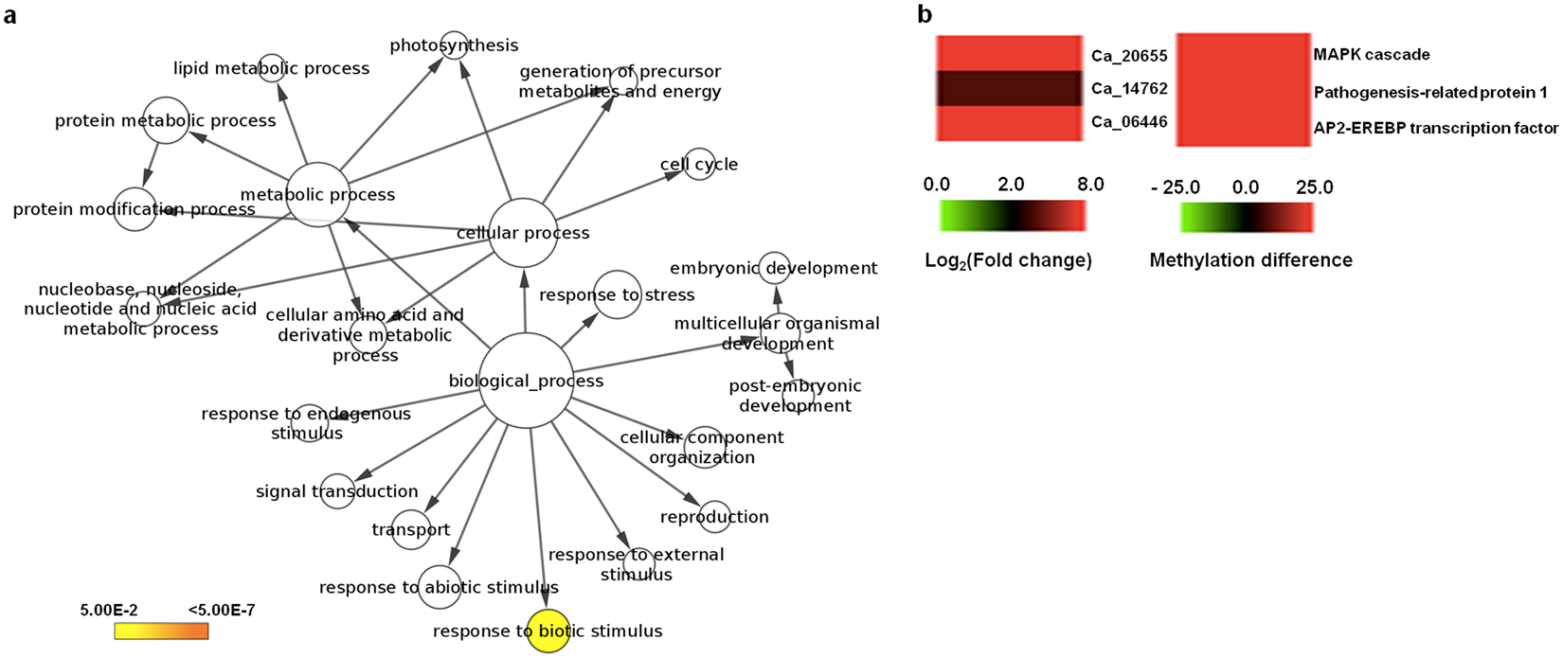
GO enrichment of hyper-methylated genes in wild chickpea and differential expression/methylation of genes involved in “response to biotic stimulus”. (**a**) GO Slim enrichment analysis of hypermethylated-DMR-associated genes from leaves of wild chickpea. The significantly enriched GO terms are highlighted in yellow color (p-value < 0.05). (**b**) Heatmaps depicting the expression pattern (left) and methylation status (right) of up-regulated genes involved in “response to biotic stimulus” in wild chickpea. Left heatmap: color scale represents fold change of genes in different organs; right heatmap: color scale denotes methylation status genes in different organs.

GO enrichment analysis of DMR-associated genes in wild chickpea revealed “response to biotic stimulus” to be significantly represented among the hypermethylated genes (Fig. 7a). As wild chickpea (PI 489777) has been reported to be *Fusarium* wilt resistant^47^, we examined the genes belonging to this GO term. Among the up-regulated genes belonging to “response to biotic stimulus” GO term, two genes harbored hypermethylated CG DMRs in the gene-body in wild chickpea (*Ca_20655* and *Ca_14762*), while the third gene (*Ca_06446*) harbored a hypermethylated CHH DMR in its promoter region (Fig. 7b). These genes were not associated with any DMRs in cultivated chickpea (except *Ca_14762*, which has a CHH DMR in its downstream region in root; Supplementary Table 3). The increased abundance of these transcripts in wild chickpea may be a result of DNA hypermethylation within gene-body, as reported previously^11^. Large-scale analysis of gene expression profiles and DNA methylomes have revealed a positive association between gene-body DNA methylation and gene expression^11^. Transcript abundance of *Ca_06446* in wild chickpea may be a consequence of methylation-specific transcription factor binding to its promoter. The promoter DMR of *Ca_06446* possesses binding sites for multiple transcription factors (ERF, C2H2, bZIP, LBD, NAC and TALE), some of which have been reported to bind methylated sequences and facilitate transcriptional up-regulation^11,43^.

### Association of smRNAs and DMRs with protein-coding genes

**Figure 8:**
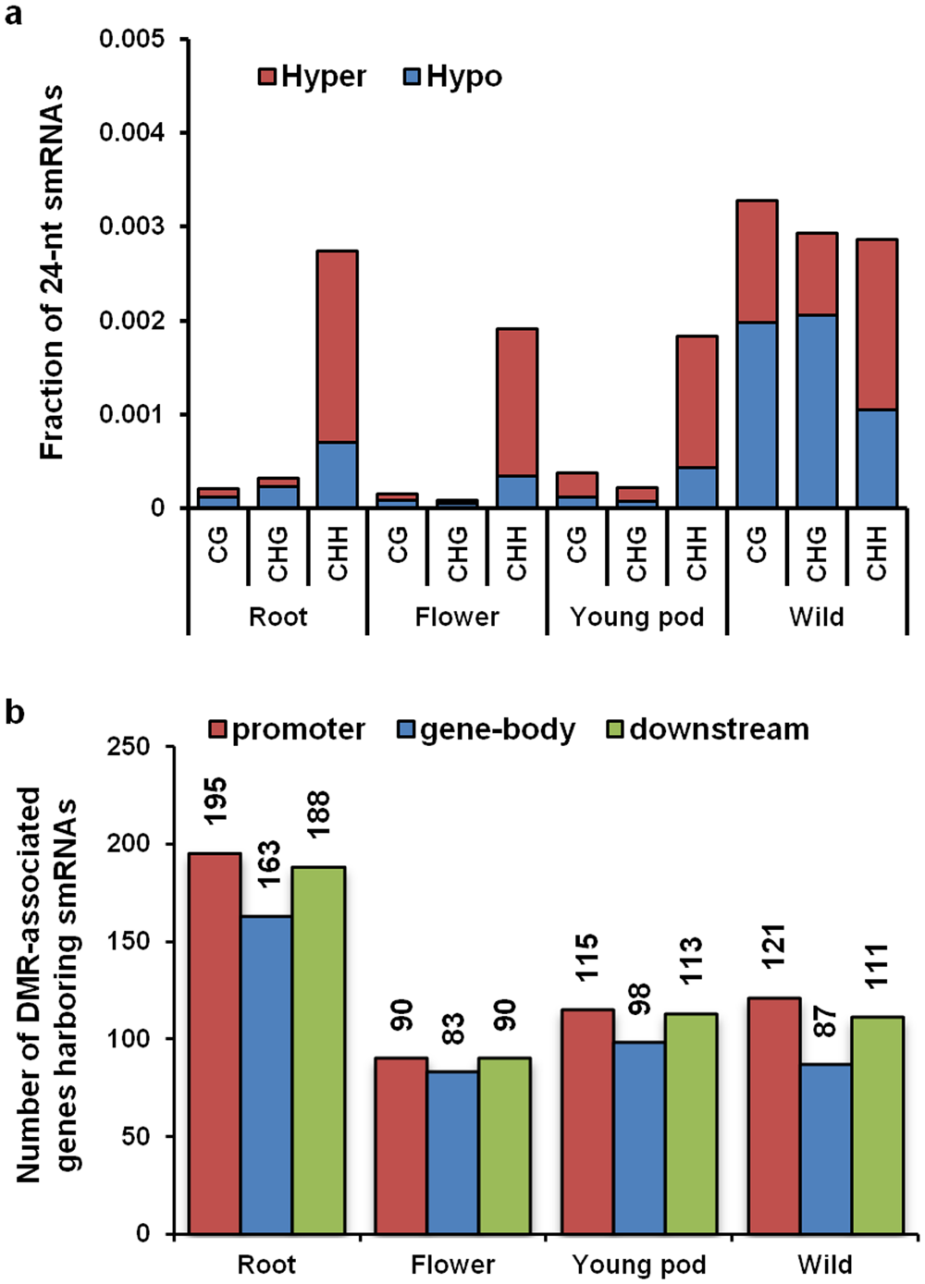
Association of smRNAs with DMRs and protein-coding genes. (**a**) Fraction of smRNAs (24 nt) associated with DMRs in each context in different organs. (**b**) Distribution of smRNAs in different regions (promoter, gene-body and downstream) of DMR-associated genes.

smRNAs contribute significantly towards epigenetic modifications in plants through RNA-directed DNA methylation pathway (RdDM)^10,48,49^. The 24-nt smRNAs recruit RdDM machinery and have been reported to modulate gene expression. We assessed 24-nt smRNAs associated with DMRs in each sample. smRNA distribution appeared to be uniform throughout each chromosome, irrespective of the methylation density (Supplementary Fig. S2). In cultivated chickpea, a large number of smRNAs were found to be associated with CHH context DMRs as compared with CG and CHG context DMRs (Fig. 8a). This is not surprising since CHH methylation is produced by the RdDM pathway^8^. To determine if genomic location of smRNAs has a role in modulating gene expression, we assessed their distribution across DMR-associated genes. For each organ, smRNAs were primarily seen to be localized within the 2Kb upstream and downstream region of the genes, while a fewer number were associated with the gene-body (Fig. 8b). These observations suggest a correlation between smRNAs and transcription factor/regulatory protein binding sites within promoters.

Majority of smRNAs were associated with hypermethylated DMRs in CHH context and with hypomethylated CG and CHG DMRs in roots and flowers (Fig. 8a). In young pod, smRNAs were primarily associated with hypermethylated DMRs in all contexts. In wild chickpea, most of the smRNAs were associated with hypermethylated DMRs in CHH context, and hypomethylated DMRs in CG/CHG context (Fig. 8a). Examination of the promoter DMRs of *Ca_19529* (from Fig. 5b) and *Ca_18733* (from Fig. 6b) revealed that multiple smRNAs originate from this region. This observation points towards possible methylation of the promoter by 24-nt smRNAs, thereby producing CHH DMRs in flowers. As explained above, the promoter DMRs of these genes possess binding sites for multiple transcription factors. Thus, it is possible that transcription factor binding to promoter DMRs of these genes may be inducing their expression in flower.

In conclusion, our analysis is the first attempt to decipher the methylome and understand the organ-specific methylation pattern in legume crop chickpea. We observed a preponderance of CG methylation within gene-body and CHH methylation within promoter/downstream regions. Gene expression was influenced by the methylation status of nearby regions, genomic location and context of associated DMRs, and 24-nt smRNAs. Promoter CHH methylation correlated positively while gene-body CG methylation correlated negatively with gene expression. Finally, association of smRNAs and TEs was observed with hypermethylated DMRs in cultivated chickpea, highlighting their role in gene expression regulation. Thus, our data puts forth novel insights in terms of methylation-dependent gene expression pattern and can facilitate the discovery of novel gene regulatory mechanisms in chickpea and other plants.

## Methods

### Plant materials and DNA extraction

The chickpea (*Cicer arietinum* L.) genotype ICC 4958 and wild chickpea (*C. reticulatum*) genotype PI 489777, were used in this study to analyze the global methylation patterns. For root tissues, plants were grown in a culture-room maintained at a maximum temperature of 22 ± 1 °C and a minimum of 12 ± 1 °C, and relative humidity of 60% under continuous light (200 *μE m*^*−*2 ^*s*^*−*1^) for 15 days. Other tissues (leaves, flowers and young pods of cultivated chickpea and leaves of wild chickpea) were harvested from the field-grown mature plants. At least three independent biological replicates were collected for each sample. DNeasy Plant Mini kit (Qiagen) was used to extract genomic DNA from all the samples. The quality and quantity of isolated genomic DNA was assessed using Nanodrop Spectrophotometer (Thermo Fisher Scientific) and Qubit Fluorometer (Life Technologies). Agarose gel electrophoresis was also performed to analyze the quality of extracted genomic DNA samples.

### Bisulphite sequencing, read alignment and detection of 5-methylcytosines

Genomic DNA isolated from each sample (pooled in equal quantity from the three independent biological replicates) was processed for bisulphite sequencing as described by *Garg et al*.^9^. The qualified libraries were sequenced on HiSeq 2000 system (Illumina) for 90 cycles in paired-end mode to achieve more than 30x genome coverage for each sample. The sequence data was filtered for adapter sequences and low-quality reads using NGS QC Toolkit (v2.3) with default parameters^50^. The quality filtered reads from each sample were aligned to the kabuli chickpea genome (V1.0)^23^ using Bismark (v0.14.3)^51^, allowing only one mismatch per seed. Only the uniquely aligned reads were retained to remove potential clonal bias due to PCR amplification. To estimate the efficiency of bisulphite conversion, alignment of all the reads was performed on the chickpea chloroplast genome as well. A bisulphite conversion efficiency of ≥99% was achieved for all the samples (Supplementary Table 1). The mapped reads on the genome were used as input in methylKit (v0.9.2)^52^ for further analysis. Identification of mCs was performed as described by Garg *et al*.^9^, with minimum read coverage of ≥5 and *P*-value ≤ 0.0001. Various analyses, such as relative distribution of mCs on the genome and various genomic features, identification of associated genes/small RNAs (smRNAs)/transposable elements (TEs) were performed using custom perl scripts.

### Identification and annotation of DMRs

To identify DMRs, methylKit (v0.9.2) package was used^52^. Leaf sample of cultivated chickpea was considered as reference. Only the Cs covered by ≥5 reads were considered for this analysis. For determination of DMRs, default parameters (tilling window approach) in methylKit were used with window size of 100 and step size of 50. Statistical significance (q-value) was determined using Fisher’s exact test followed by Sliding Linear Model (SLIM) correction. A bin was considered as a DMR, if it contained a minimum of 5 cytosines with total methylation difference ≥25% (with q-value ≤ 0.01) as compared with the reference sample. Customized R script using GenomicRanges was used for DMR annotation. GO enrichment analysis was performed using BiNGO plugin of Cytoscape^53^.

### RNA sequencing and data analysis

Total RNA was extracted from all samples using TRI Reagent (Sigma Life Science, USA) according to the manufacturer’s instructions. RNA quantification was performed using Nanodrop Spectrophotometer, and quality was assessed using Agilent Bioanalyzer (Agilent Technologies, Singapore), as described previously^9^. cDNA libraries were prepared for each sample, and sequencing was performed on Illumina HiSeq platform to generate 51 bp single-end (leaf, flower and young pod), 46 bp single-end (root) or 100 bp paired-end (leaf of wild chickpea) reads. The FASTQ files obtained were pre-processed using NGS QC Toolkit^50^ to remove adapters and low-quality reads. An average of 32.023 million high-quality reads were obtained from each sample, of which 99% mapped to the chickpea genome using TopHat2 pipeline (v2.0.0)^54^. To analyze gene expression, a consensus reference-guided assembly of the transcriptome data from all samples was generated using Cufflinks (v2.0) and differential expression of genes was determined by Cuffdiff as described^9^. Only the genes exhibiting significant difference (absolute fold change ≥2, with P-value ≤ 0.05) were considered as differentially expressed.

### Small RNA sequencing and data analysis

Small RNA data for four samples of ICC 4958 (leaves, roots, flowers, and young pods) were taken from our previous study^24^. Small RNA library from leaves of wild chickpea PI 489777 was prepared using TrueSeq Small RNA Sample Prep Kit (Illumina Technologies), according to the manufacturer’s instructions and sequenced on Illumina platform. Data obtained in FASTQ files was pre-processed to remove low-quality reads, as described previously^24^. Unique reads from each sample were mapped to the chickpea genome using Bowtie^55^. Only the non-redundant set of uniquely mapped reads (small RNA) of 24-nt length were used for analysis.

### Data availability

BS-seq, RNA-seq and small RNA-seq data generated in the present study have been deposited in the Gene Expression Omnibus under the series accession numbers GSE103575, GSE103561 and GSE103571, respectively.

## Electronic supplementary material

Supplementary Information

Table S2

Table S3
