# Supplementary material for: Genome-wide bisulphite-sequencing reveals organ-specific methylation patterns in chickpea
Source: Sci Rep. 2018 Jun 26;8:9704. doi: 10.1038/s41598-018-27979-w (PMC6018830; doi:10.1038/s41598-018-27979-w)
Supplement: Supplementary file 1 — Supplementary Information [file 41598_2018_27979_MOESM1_ESM.pdf]

Supplementary Information

Subject Area: Plant Biology

+Correspondence and requests for material should be addressed to R.G. (rohini.garg@snu.edu.in**).**

Genome-wide bisulphite-sequencing reveals organ-specific methylation patterns in chickpea

Himanshi Bhatia1*, Niraj Khemka1*, Mukesh Jain1,2 Rohini Garg3+

1 School of Computational and Integrative Sciences, Jawaharlal Nehru University, New Delhi – 110067, India

2 National Institute of Plant Genome Research (NIPGR), Aruna Asaf Ali Marg, New Delhi - 110067, India.

3 Department of Life Sciences, School of Natural Sciences, Shiv Nadar University, Gautam Buddha Nagar, Uttar Pradesh - 201314, India

*These authors contributed equally to this work.

Supplementary Figure S1. DNA methylation level in each sequence context (CG, CHG and CHH) in various organs. Line graph depicts the fraction of mCs with different levels of methylation.

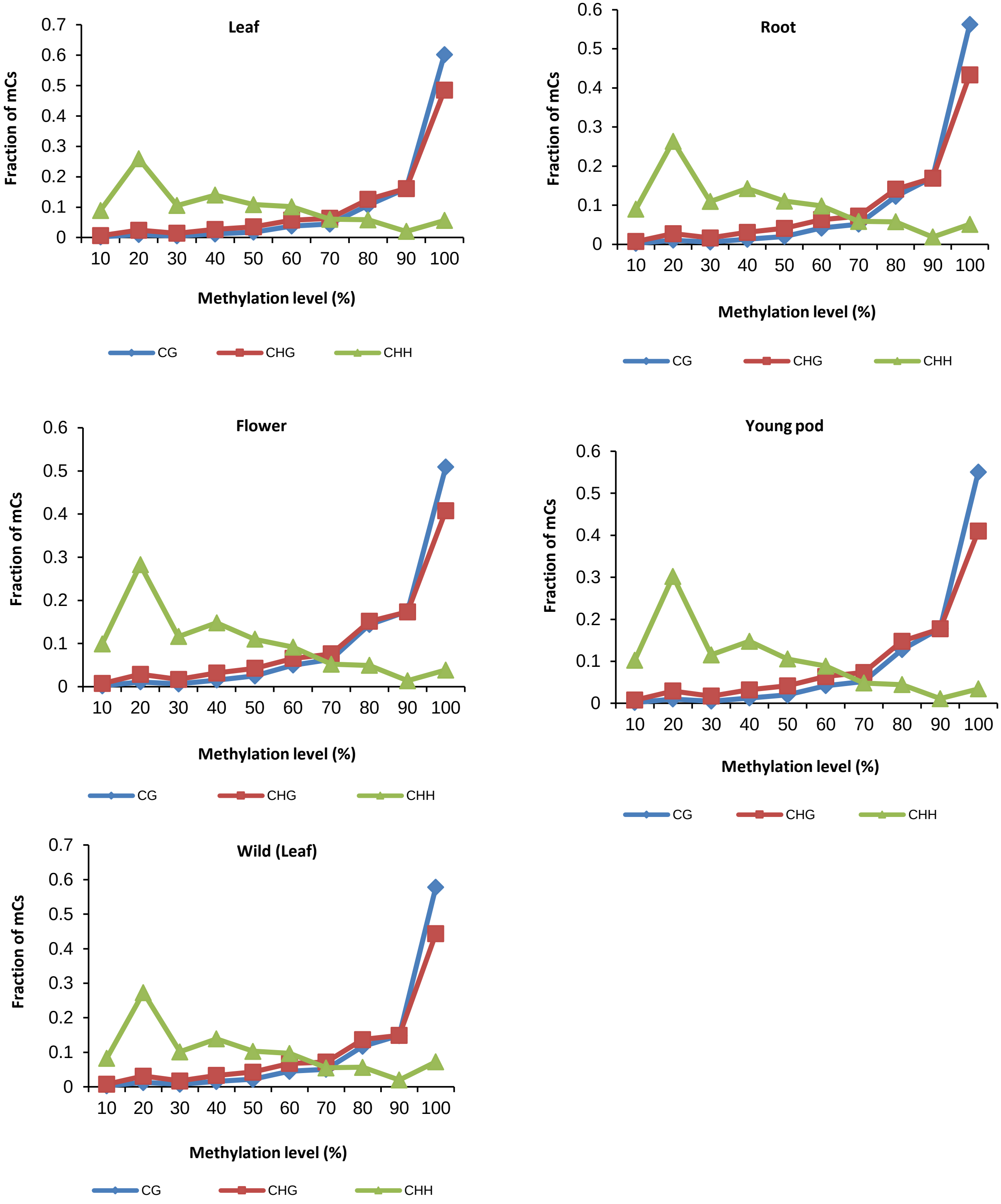

Supplementary Figure S2. Circos plots depicting chromosome-wise distribution of CG (I), CHG (II), and CHH (III) methylation, and density of genes (IV), TEs (V), and 24 nt smRNAs (VI) for various organs. Green and red peaks denote methylation density at sense and anti-sense strand, respectively.

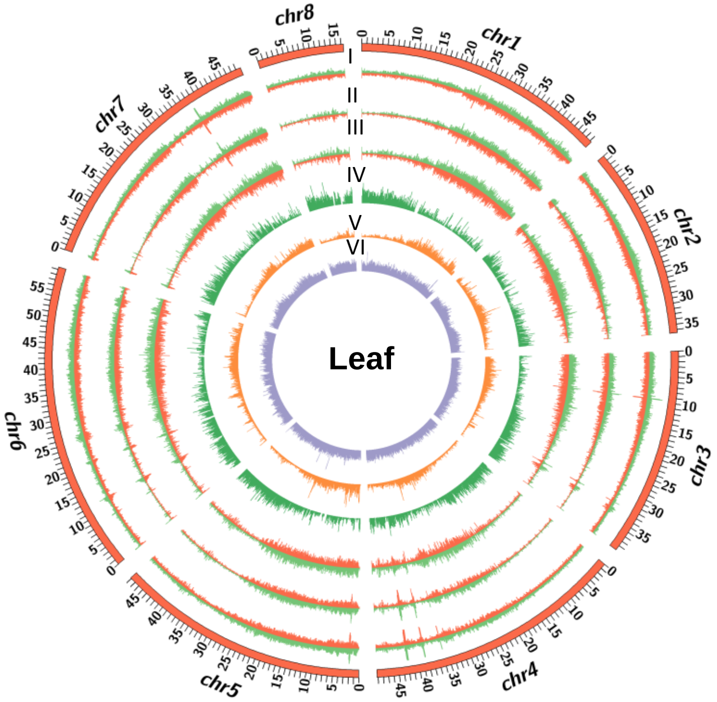

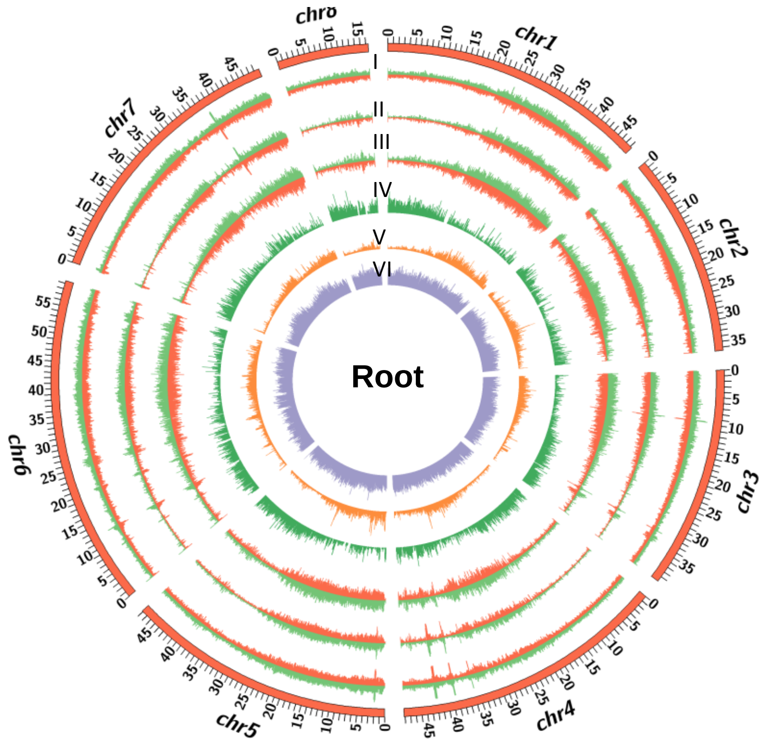

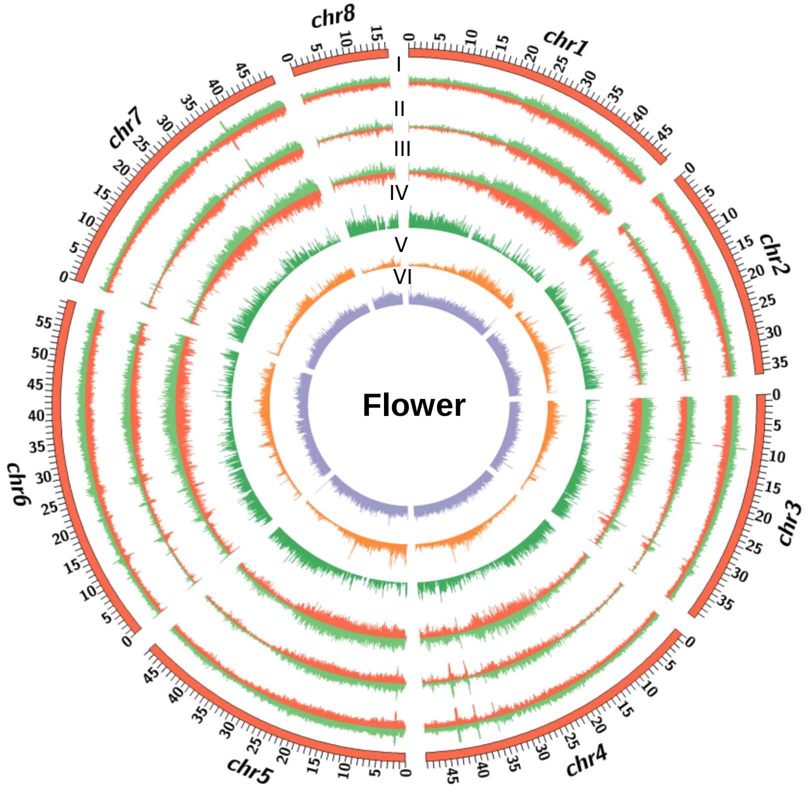
chr1
chr2
chr3
chr4
chr5
chr6
chr7
chr8
I
II
III
IV
V
VI
Flower

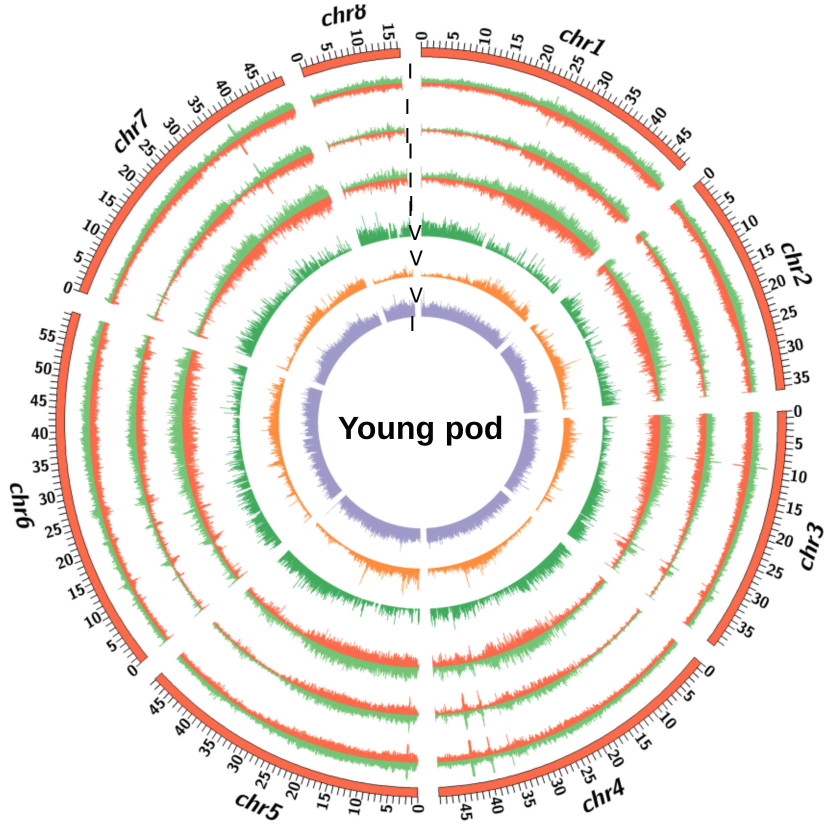
chr1
chr2
chr3
chr4
chr5
chr6
chr7
chr8
Young pod

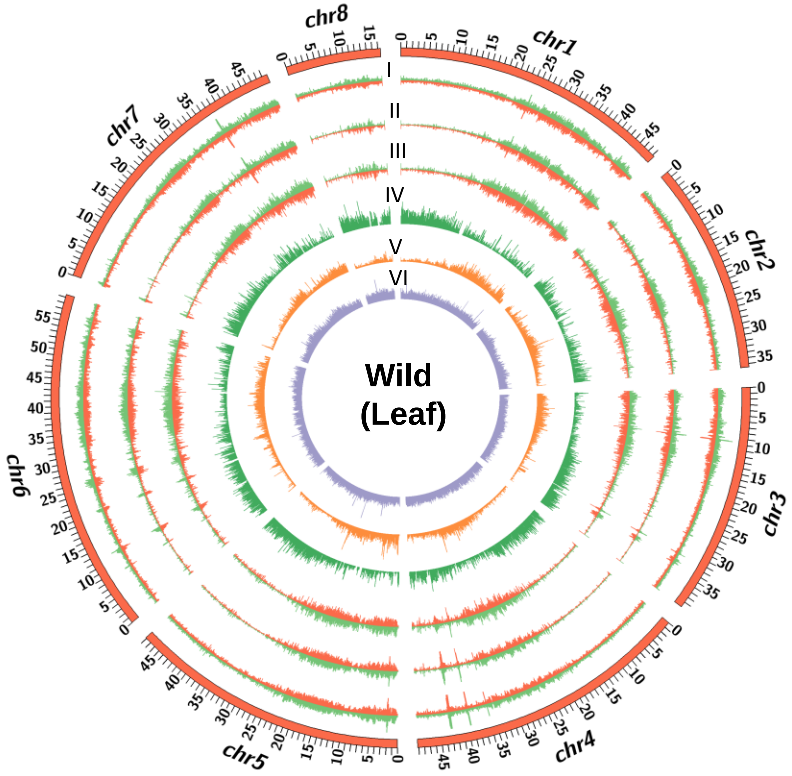
chr1
chr2
chr3
chr4
chr5
chr6
chr7
chr8
I
II
III
IV
V
VI
Wild
(Leaf)

Supplementary Figure S3. Expression analysis of genes from various organs. **(a)** Total number of significantly expressed genes in various organs. **(b)** Number of DMR-associated genes in various organs. **(c)** Number of up- and down-regulated DMR-associated genes in various organs.

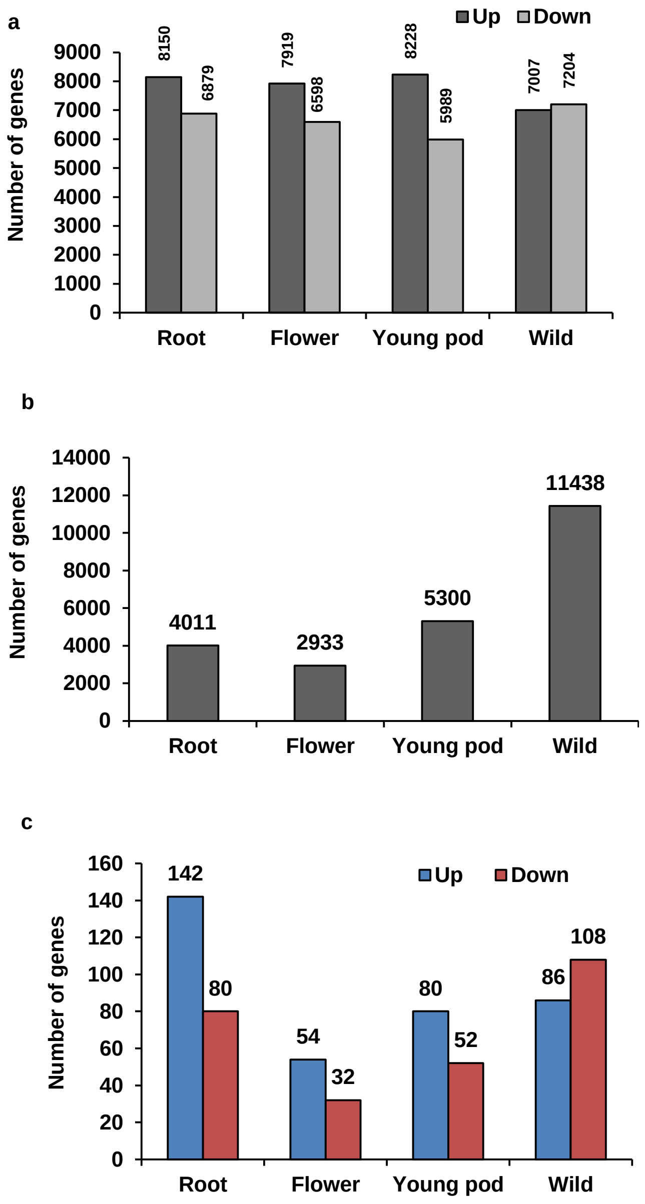

Supplementary Table S1. Summary of bisulfite sequencing data generated, genome alignment and methylcytosines for different tissues.

	Cultivated chickpea (ICC 4958)				Wild chickpea (PI 489777)
	Leaf	Root	Flower	Young pod	Leaf
Total read pairs analysed	108398958	108194029	107594656	108277587	108013264
Uniquely mapped read pairs	52077671	56341156	58650195	59781863	46142471
Mapping efficiency	48.0%	52.1%	54.5%	55.2%	42.7%
Genome coverage	86.52%	88.23%	88.35%	88.54%	80.85%
Bisulfite conversion efficiency	99.6%	99.2%	99.46%	99.6%	99.6%
Error rate	0.400%	0.809%	0.539%	0.388%	0.387%
Total Cs analysed	118054976	127473299	130118443	130152150	115279174
Total methylated Cs	17568160	20600295	21524658	21736643	15303135
Methylated Cs	14.88%	16.16%	16.54%	16.70%	13.27%

Supplementary Table S2. List of DMR-associated genes in different organs.

(*Provided as separate MS Excel file)*

Supplementary Table S3. List of genes with CG context DMRs in gene-body and CHH context DMRs in flanking region in different organs.

(*Provided as separate MS Excel file)*
